# A Scoping Review of Inborn Errors of Metabolism Causing Progressive Intellectual and Neurologic Deterioration (PIND)

**DOI:** 10.3389/fneur.2019.01369

**Published:** 2020-02-18

**Authors:** Hilde A. G. Warmerdam, Elise A. Termeulen-Ferreira, Laura A. Tseng, Jessica Y. Lee, Agnies M. van Eeghen, Carlos R. Ferreira, Clara D. M. van Karnebeek

**Affiliations:** ^1^Department of Pediatrics, Emma Children's Hospital, Amsterdam Gastroenterology and Metabolism, Amsterdam University Medical Centres, University of Amsterdam, Amsterdam, Netherlands; ^2^Centre for Molecular Medicine and Therapeutics, BC Children's Hospital Research Institute, University of British Columbia, Vancouver, BC, Canada; ^3^'s Heeren Loo Care Group, Amsterdam, Netherlands; ^4^National Human Genome Research Institute, National Institutes of Health, Bethesda, MD, United States; ^5^Department of Pediatrics, Radboud Centre for Mitochondrial Medicine, Radboud University Medical Centre, Nijmegen, Netherlands

**Keywords:** PIND, inherited metabolic diseases, neurodegeneration, dementia, loss of skills, genetic, treatment, diagnosis

## Abstract

**Background:** Progressive intellectual and neurological deterioration (PIND) is a rare but severe childhood disorder characterized by loss of intellectual or developmental abilities, and requires quick diagnosis to ensure timely treatment to prevent possible irreversible neurological damage. Inborn errors of metabolism (IEMs) constitute a group of more than 1,000 monogenic conditions in which the impairment of a biochemical pathway is intrinsic to the pathophysiology of the disease, resulting in either accumulation of toxic metabolites and/or shortage of energy and building blocks for the cells. Many IEMs are amenable to treatment with the potential to improve outcomes. With this literature review we aim to create an overview of IEMs presenting with PIND in children, to aid clinicians in accelerating the diagnostic process.

**Methods:** We performed a PubMed search on IEMs presenting with PIND in individuals aged 0–18 years. We applied stringent selection criteria and subsequently derived information on encoding genes, pathways, clinical and biochemical signs and diagnostic tests from IEMbase (www.iembase.org) and other sources.

**Results:** The PubMed search resulted in a total of 2,152 articles and a review of references added another 19 articles. After applying our selection criteria, a total of 85 IEMs presenting with PIND remained, of which 57 IEMs were reported in multiple unrelated cases and 28 in single families. For 44 IEMs (52%) diagnosis can be achieved through generally accessible metabolic blood and urine screening tests; the remainder requires enzymatic and/or genetic testing. Treatment targeting the underlying pathophysiology is available for 35 IEMs (41%). All treatment strategies are reported to achieve stabilization of deterioration, and a subset improved seizure control and/or neurodevelopment.

**Conclusions:** We present the first comprehensive overview of IEMs presenting with PIND, and provide a structured approach to diagnosis and overview of treatability. Clearly IEMs constitute the largest group of genetic PIND conditions and have the advantage of detectable biomarkers as well as amenability to treatment. Thus, the clinician should keep IEMs at the forefront of the diagnostic workup of a child with PIND. With the ongoing discovery of new IEMs, expanded phenotypes, and novel treatment strategies, continuous updates to this work will be required.

## Introduction

Progressive intellectual and neurological deterioration (PIND) in children is defined as “progressive deterioration for more than 3 months with loss of already attained intellectual or developmental abilities and development of abnormal neurological signs” (1–6). Initial psychomotor development might be delayed, but this is not required. PIND is a rare, yet alarming phenomenon. It will urge every pediatrician to find a diagnosis and quickly start treatment in order to halt or even reverse the intellectual decline in the patient. Additionally, this will provide an opportunity for accurate prognostication and genetic counseling for the family.

However, evaluation is extremely challenging given the immense heterogeneity of underlying disorders, varying from genetic causes such as intellectual disability syndromes (e.g., Rett disease, MIM#312750), neurodegenerative repeat expansion diseases, chromosome breakage diseases as well as inborn errors of metabolism, to environmental causes such as infectious encephalitis, traumatic brain injury, anoxic brain injury, or immune mediated conditions such as anti-MNDA receptor encephalitis (8, 9). Inborn errors of metabolism (IEMs) constitute a well-known cause of PIND and intellectual developmental disabilities in general; examples include neuronal ceroid lipofuscinosis (NCL), Niemann-Pick disease type C (NPC), and different types of mucopolysaccharidosis (MPS) (8–10). Nonetheless, a complete overview of IEMs causing PIND remains unavailable in the current literature, and this group of single gene disorders is not commonly screened for with biochemical urine/blood tests, despite increasing opportunities to causally treat and improve prognosis. Treatment strategies include diet, vitamin supplements, medications, and hematopoietic stem cell transplantation (HSCT) to enzyme replacement or gene therapy.

Here we aim to present an up-to-date literature review of all known IEMs reported with a clinical presentation of PIND. With the information provided about phenotypic features, diagnostic tests and therapeutic modalities, we hope to aid the clinician in the efficient identification and management of IEMs in children presenting with PIND.

## Methods

### Search Strategy and Defining PIND and IEMs

We searched PubMed with terms encompassing and describing our definitions of PIND and IEMs to ensure the search yielded articles meeting our search query, i.e., IEMs presenting with PIND in children (Table 1).

**Table 1:** Terms used for search strategy in Pubmed.

Neurocognitive regression	Progressive intellectual and neurologic deterioration, PIND, neurologic/neurocognitive/psychomo tor/mental/cognitive/developmental/intellectual regression/deterioration/decline, developmental arrest, dementia, loss of skill(s), loss of milestone(s), neurobehavioral signs and symptoms, loss of (intellectual) abilities, disintegrative/neurodegenerative disease/disorder.
Inborn errors of metabolism	Inborn/inherited errors of metabolism, inborn/inherited/genetic metabolic/neurometabolic/ metabolic brain disorder/disease, IEMs
Age group	Newborn, infant, child, pediatric,
(birth-18 years)	adolescent.

Inborn metabolic diseases were re-defined in 2015 by Morava et al. as “a disruption of a metabolic pathway irrespective of whether it is associated with abnormalities in biochemical laboratory tests” (7). Recently Ferreira et al. proposed a new nosology using this re-definition of IEMs, the inclusion criteria of which are shown in Table 2 (8). These criteria were subsequently used to define IEMs in this review.

**Table 2:** Criteria inborn errors of metabolism by Ferreira et al. (8).

Disruption of a metabolic pathway is considered necessary and sufficient for inclusion Regardless of laboratory abnormalities in standard biochemical tests Regardless of association with clinical manifestations of disease (unless defect is universal to all humans) Severity alone is not considered sufficient for separation into different entries when a single gene product is involved A different pathomechanism is considered necessary for separation into different entries when a single gene product is involved, regardless of the mode of inheritance The involvement of different gene products is considered sufficient for separation into different entries, even if the phenotype is similar The error must have been reported in more than a single family, and the involvement of the gene product must haven well characterized on an enzymatic or molecular level

Table 3 shows the definition of PIND as used for this review and is based on the definition of UK (1–4), Canadian (5) and Australian (6) research groups exploring which disorders present with symptoms consistent with PIND (1–6). Our definition of PIND includes progressive deterioration with loss of already attained intellectual and developmental abilities and development of neurological signs including epilepsy. Deterioration through loss of intellectual or developmental abilities may be present in children with previously normal or delayed development. No distinction is made in this review.

**Table 3:** Definition PIND.

**Children aged 0−18 years at onset of symptoms who fulfill the following criteria:**
• Progressive deterioration during 3 months
• Loss of already attained intellectual or developmental abilities
• Development of neurological signs including epilepsy
• No (static) loss of skill due to:
- Encephalitis
- Head injury
- Near drowning

### Expert Opinion

If the literature was inconclusive, individual experts were consulted for their input on specific (groups) of IEMs and requested to provide an evidence- or expert-based opinion on the presence or absence of PIND as a presenting feature. If literature of sufficient quality meeting our criteria was available, then the IEM was included in this list.

### Literature Search and Selection

We searched PubMed for all articles published between 1953 and October 2018 with the aim of establishing which inborn errors of metabolism cause neurocognitive regression in children aged 0–18 years.

**Figure 1:**
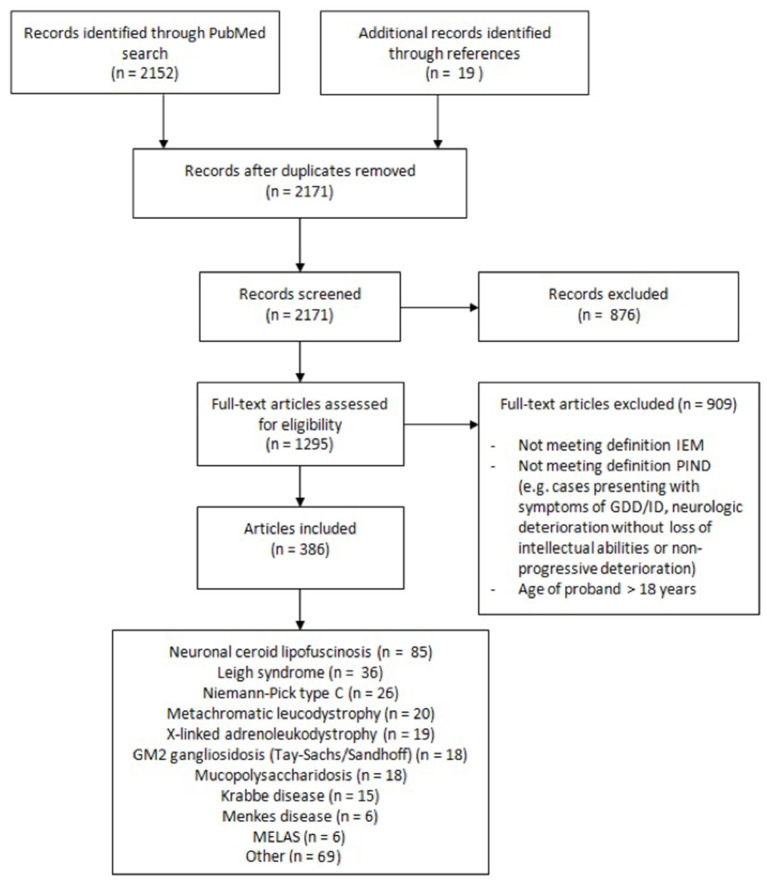
Flow diagram showing the different phases of the literature selection process.

A flow diagram representing the different phases of the selection process is shown in Figure 1. Selection of articles according to inclusion and exclusion criteria was performed with the use of Rayyan (https://rayyan.qcri.org), a web and mobile app for systematic reviews (9). Exclusion criteria were as followed: (1) not meeting definitions of IEM and/or PIND; (2) IEM not manifesting with PIND; (3) IEM presenting with completely reversible intellectual and neurologic deterioration, e.g., during (febrile) disease; (4) IEM presenting with neurologic or movement disorders without intellectual deterioration; (5) IEM presenting solely with developmental delay; (6) age of proband(s) over 18 years; (7) animal studies; (8) no (English or Dutch) full text available.

We compared our list of selected IEMs with those of PIND research groups and added IEMs of the latter if these met our selection criteria. Also, we consulted experts for their opinion.

Articles publishing on IEMs causing symptoms meeting the definition of PIND were further pursued. When meeting all criteria, the IEMs were categorized in nine groups using the proposed nosology by Ferreira et al.: A. Disorders of nitrogen-containing compounds; B. Disorders of vitamins, cofactors, metals and minerals; C. Disorders of carbohydrates; D. Mitochondrial disorders of energy metabolism; E. Disorders of lipids; F. Disorders of tetrapyrroles; G. Storage disorders; H. Disorders of peroxisomes and oxalate; and I. Congenital disorders of glycosylation (8).

### Disease Information

If the literature search yielded multiple articles for a specific disorder (e.g., neuronal ceroid lipofuscinosis and Leigh syndrome), databases such as GeneReviews, OMIM, OMMBID and IEMbase were consulted for more concise summaries or reviews.

IEMbase (www.iembase.org), an online IEM knowledgebase, was searched for information on each IEM such as encoding genes, pathways, clinical symptoms, biochemical signs, and diagnostic tests. Other databases such as Online Mendelian Inheritance in Man (OMIM)^1^, GeneReviews (11) and the Online Metabolic and Molecular Bases of Inherited Disease (OMMBID) (12) were consulted if additional information was required.

### Diagnostic Strategies

To facilitate a practical guide for biochemical and genetic diagnosis, we assessed which tests are necessary to diagnose each of the conditions. Accordingly, we grouped the diseases into IEMs diagnosed via “metabolic screening tests” vs. IEMs diagnosed via “enzymatic and/or genetic testing”. As screening tests we defined biochemical tests in blood and urine for IEMs with reliable biomarkers, generally available in biochemical laboratories in most developed countries.

### Therapeutic Modalities

Information about treatment was extracted from two articles on treatable intellectual disabilities, i.e., IEMs causing global developmental delay (DD)/intellectual disability (ID) which are amenable to causal treatment (13, 14) (this information is available via an online digital tool, www.treatable-id.org) (15). A treatable IEM is defined as the availability of a particular therapeutic modality capable of preventing or improving the DD/ID phenotype, or halting/slowing neurocognitive decline with acceptable adverse effects, i.e., positively influencing the “outcome measures”. Therapeutic modalities include dietary restriction/supplementation, cofactor or vitamin supplementation, substrate inhibition (small molecule), substrate reduction, enzyme replacement, bone marrow and hematopoietic stem cell transplant and gene therapy. Outcome measure/effect are defined as primary = IQ, developmental testing score/performance, survival; secondary = epilepsy, behavior, psychiatric, neurological deficit (e.g., movement disorder), neuro-imaging, or systemic symptoms influencing developmental/cognitive performance (e.g., ichthyosis, liver disease). The level of evidence, i.e., the quality of the evidence describing the beneficial effect of each therapeutic modality, was ranked 1–5 according to the Center of Evidence Based Medicine (www.cebm.net).

## Results

### Literature Yield

Our Pubmed search yielded 2,152 articles. After applying inclusion and exclusion criteria we identified a total of 79 IEMs presenting with PIND. Of these 79 IEMs, 55 (70%) presented with PIND in multiple cases and in multiple families, while the remaining 24 IEMs presented with PIND in single cases or single families (Supplemental Table S1).

We added four IEMs to Supplemental Table S1 that did not emerge from our literature search but rather were reported in articles by PIND research groups, and agreed upon by our expert colleagues as IEMs to present with PIND. Based on expert opinion, we included two more IEMs, mitochondrial DNA polymerase-gamma catalytic subunit (POLG) deficiency, and folate receptor-alfa deficiency, resulting in a total of 85 IEMs presenting with PIND.

Taking the total of 85 IEMs presenting with PIND, storage disorders (*n* = 34/85, 40%) represented the largest category. The other IEMs were classified as follows: nitrogen-containing compounds (*n* = 15); disorders of vitamins, cofactors, metals and minerals (*n* = 15); disorders of carbohydrates (*n* = 1); mitochondrial disorders of energy metabolism (*n* = 14); disorders of lipids (*n* = 2); disorders of tetrapyrroles (*n* = 1); disorders of peroxisomes and oxalate (*n* = 2); and congenital disorders of glycosylation (*n* = 1).

Neurologic and systemic symptoms registered in IEMBase are shown in Supplemental Table S1 Besides PIND, these disorders present with a variety of neurologic symptoms, most commonly: seizures (or epilepsy, convulsions, 67 IEMs, 79%), global developmental delay/intellectual disability (GDD/ID, 33 IEMs, 39%), and ataxia (54 IEMs, 63%), but hypotonia, nystagmus, MRI abnormalities, loss of vision and loss of hearing may also be present. Non-neurologic symptoms vary widely from vomiting, retinopathy and hepatosplenomegaly to psychiatric and behavioral disorders.

The case of Leigh syndrome (MIM#256000), one of the IEMs associated with PIND, deserves special mention. Leigh syndrome is a progressive neurodegenerative disorder with developmental regression, usually between ages 3 and 12 months, due to mitochondrial oxidative phosphorylation defects. Typical MRI abnormalities include symmetrical lesions in the basal ganglia or brainstem. This syndrome is not associated with mutations in a single gene, but is caused by many different gene defects; currently there are 178 genes associated with Leigh syndrome in the Leigh Map (available at vmh.uni.lu/#leighmap) (16).

### Diagnostic Strategies

**Figure 2:**
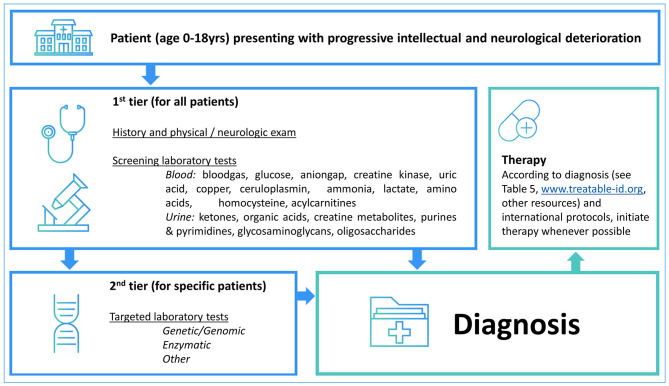
Two-tiered diagnostic biochemical and genetic algorithm. The potential yield of the 1st tier laboratory tests is 52% (*n* = 44 IEMs) while the remaining 41 IEMs are identified via the 2nd tier tests. Exome/genome sequencing can be initiated according to the local availability and clinical practice as well as specialists' insights.

Table 4 summarizes the diagnostic methods required for identification of IEMs presenting with PIND. A total of 44 IEMs can be identified through metabolic screening tests in blood and urine, 14 IEMs require enzymatic analysis, while for the remaining 30 IEMs, reliable biomarkers are lacking and genetic testing is obligatory. This information is summarized in Figure 2, i.e., a two-tiered diagnostic algorithm comprising biochemical and genetic testing. Exome/genome sequencing should be initiated according to the insight of the clinician. Finally, 7 IEMs associated with PIND are included in newborn screening (NBS) panels in various countries (Supplemental Table S1).

**Table 4:** Diagnostic tests.

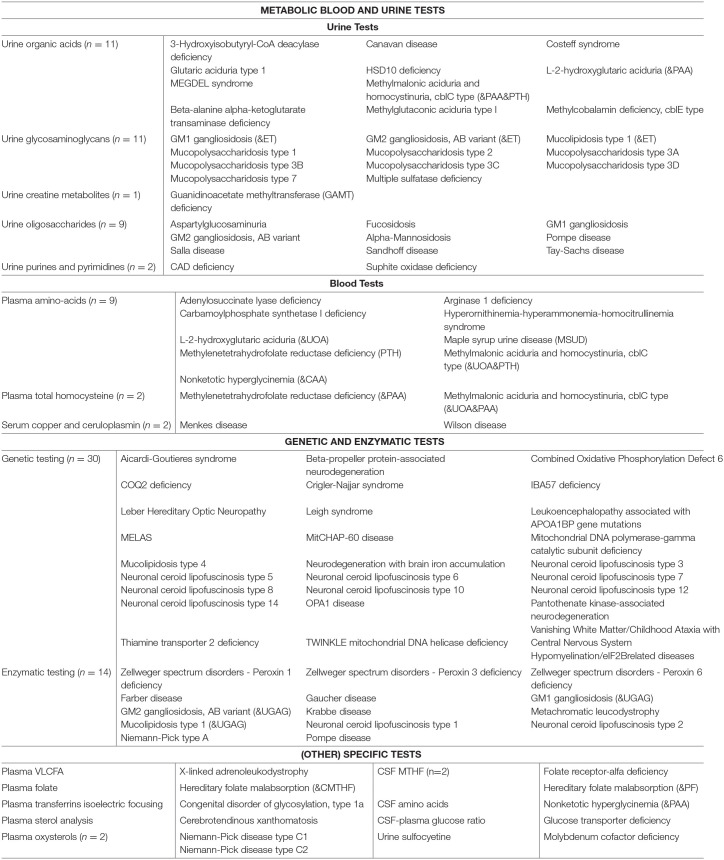

### Therapeutic Modalities

Table 5 provides an overview of all IEMs presenting with PIND for which causal treatment is available, totaling 35 IEMs (41%).

**Table 5:** Therapeutic modalities for IEMs causing PIND.

**IEM name**	**Therapeutic modality (-ies)**	**Level of evidence**	**Treatment effect**	**References**
α-mannosidosis	HSCT	4/5	Stabilizes clinical deterioration	Treatable ID
Aspartylglucosaminuria	HSCT	4/5	Stabilizes clinical deterioration	Treatable ID
Arginase 1 deficiency	Dietary protein restriction, arginine supplement, sodium benzoate, phenylbutyrate	2b	Prevents metabolic decompensation; stabilizes clinical deterioration; improves behavior, seizure control, neurological and systemic manifestations	Treatable ID
Biotinidase deficiency	Biotin supplements	2c	Improves psychomotor development/IQ, neurological and systemic manifestations	Treatable ID
CAD deficiency	Uridine supplements	4	Seizure control, stabilizes clinical deterioration, improves psychomotor development/IQ	Koch et al. (31)
Carbamoylphosphate synthetase I deficiency*	Dietary protein restriction, arginine supplement, sodium benzoate, phenylbutyrate	2b	Prevents metabolic decompensation; stabilizes clinical deterioration; improves behavior, seizure control, neurological and systemic manifestations	Treatable ID
Cerebral folate receptor-α deficiency	Folinic Acid	4	Stabilizes clinical deterioration; improves psychomotor development/IQ; seizure control and neurological manifestations	Treatable ID
Cerebrotendinous xanthomatosis	Chenodeoxycholic Acid, HMG Reductase Inhibitor	4	Stabilizes clinical deterioration, improves behavior, neurological and systemic manifestations	Treatable ID
COQ2 deficiency	CoQ supplements	4	Improves neurological manfestation and seizure control	Treatable ID
Gaucher disease (type III)	HSCT	4/5	Stabilizes clinical deterioration, improves systemic manifestations	Treatable ID
Glucose transporter deficiency	Ketogenic or analog diets	2b	Improves seizure control, improves neurological manifestations, improves psychomotor development/IQ	Daci et al. (32)
Guanidinoacetate methyltransferase (GAMT) deficiency	Arginine restriction, Creatine and Ornithine supplements	4	Stabilizes clinical deterioration; improves behavior, seizure control and neurological manifestations	Treatable ID
HSD10 deficiency	Avoid fasting, Sick day management, Isoleucine restricted diet	5	Prevents metabolic decompensation	Treatable ID
Hyperornithinemia-hyperammonemiahomocitrullinemia syndrome	Dietary protein restriction, Ornithine supplement, sodium benzoate, phenylacetate	4	Prevents metabolic decompensation; stabilizes clinical deterioration; improves behavior, seizure control, neurological and systemic manifestations	Treatable ID
Krabbe disease	HSCT	2a	Stabilizes clinical deterioration	Escolar et al. (33)
l.o. Glutaric aciduria type 1	Lysine Restriction, Carnitine supplements	2c	Prevents metabolic decompensation; stabilizes clinical deterioration; improves neurological and systemic manifestations	Treatable ID
l.o. Metachromatic leucodystrophy	HSCT	4/5	Stabilizes clinical deterioration	Treatable ID
Maple syrup urine disease (variant)	Amino acids, Avoid fasting	4	Stabilizes clinical deterioration; prevents metabolic decompensation; improves behavior	Treatable ID
Menkes disease occipital horn syndrome	Copper Histidine	4	Stabilizes clinical deterioration	Treatable ID
Methylcobalamin deficiency, cblE type	Hydroxy-/Methylcobalamin, Betaine	4	Prevents metabolic decompensation, stabilizes clinical deterioration; improves systemic manifestations	Treatable ID
Methylenetetrahydrofolate reductase deficiency	Betaine supplements, +/– Folate, Carnitine, Methionine supplements	4	Prevents metabolic decompensation; stabilizes clinical deterioration; improves systemic manifestations	Treatable ID
Methylglutaconic aciduria type I	Carnitine supplements, Avoid fasting, Sick day management	5	Prevents metabolic decompensation	Treatable ID
Methylmalonic aciduria and homocystinuria, cblC type	Hydroxocobalamin	4	Prevents metabolic decompensation; stabilizes clinical deterioration; improves systemic manifestations	Treatable ID
Mitochondrial Myopathy, Encephalopathy, Lactic Acidosis and Stroke-like episodes (MELAS)	Arginine supplements	4/5	Prevents metabolic decompensation, stabilizes clinical deterioration, improves seizure control & neurological manifestations	Treatable ID
Molybdenum cofactor deficiency	Cyclic pyranopterin monophosphate (cPMP) supplements	2b	Stabilizes clinical deterioration; improves neurological manifestations	Schwahn et al. (34)
Mucopolysaccharidosis type 1 (MPS I)	HSCT	1c	Stabilizes clinical deterioration; improves systemic manifestations	Treatable ID
Mucopolysaccharidosis type 2 (MPS II)	HSCT	4/5	Stabilizes clinical deterioration; improves systemic manifestations	Treatable ID
Mucopolysaccharidosis type 3A (MPS IIIA)	HSCT	4/5	Stabilizes clinical deterioration	Treatable ID
Mucopolysaccharidosis type 3B (MPS IIIB)	HSCT	4/5	Stabilizes clinical deterioration	Treatable ID
Mucopolysaccharidosis type 3C (MPS IIIC)	HSCT	4/5	Stabilizes clinical deterioration	Treatable ID
Mucopolysaccharidosis type 3D (MPS IIID)	HSCT	4/5	Stabilizes clinical deterioration	Treatable ID
Mucopolysaccharidosis type 7 (MPS VII)	HSCT	4/5	Stabilizes clinical deterioration	Treatable ID
Neuronal ceroid lipofuscinosis type 2	Enzyme replacement therapy	1c	Stabilizes clinical deterioration	Geraets et al. (35)
Niemann-Pick disease type C1	Miglustat	1b	Stabilizes clinical deterioration; improves neurological manifestations	Treatable ID
Niemann-Pick disease type C2	Miglustat	1b	Stabilizes clinical deterioration; improves neurological manifestations	Treatable ID

Legend: l.o., Late onset.

HSCT, Haematologic stem cell transplantation.

*Levels of evidence (source www.cebm.net): 1a. Systematic review of randomized controlled trials (RCTs), 1b. Individual RCT, 1c. All or none. 2a. Systematic review of cohort studies, 2b. Individual cohort study, 2c. “Outcomes” research, 3. Systematic review of case-control studies, 4.Individual case-control study or case-series/report, 4/5. Single case report, 5. Expert opinion without critical appraisal*.

The main effect of therapy is stabilization of clinical deterioration, as this effect is reported for every registered therapeutic modality. Other reported effects include improvement of seizure control (*n* = 9); behavior (*n* = 2); and neurological and/or systemic manifestations (*n* = 19).

The level of evidence for these therapies varies; for the majority the level of evidence is 4 (case series) to 5 (single case report/expert opinion) (*n* = 24, 69%). For 4 therapeutic strategies (11%), the level of evidence is 1 including: HSCT in MPS I, miglustat treatment in Niemann-Pick type C1 and C2, enzyme replacement therapy in neuronal ceroid lipofuscinosis type 2, and HSCT in X-linked adrenoleukodystrophy.

## Discussion

Here we present, for the first time, a comprehensive list of IEMs presenting with PIND, based on a PubMed search of relevant literature. The total number (*n* = 85) is higher than previously estimated, although prevalence remains unknown in the PIND and general population. Several groups have analyzed and published data on the etiology and epidemiology of PIND while researching childhood dementia, progressive deterioration, or prevalence of variant Creutzfeld-Jacob disease in children. An overview of the reported presence of inborn errors of metabolism in children with PIND is shown in Table 6. These studies show varying causes of PIND and report that 62.5–75.0% of all PIND cases are caused by IEMs. In the surveillance reports from the large cohort of the UK PIND research group, IEMs represent 82.3–88.2% of the most common diagnoses (1, 4). These numbers indicate that IEMs represent a significant proportion of the diagnoses reported in children presenting with PIND.

**Table 6:** Overview of articles reporting the identified etiology of PIND, overall and specifically the IEMs.

	**PIND cases**	**Etiology known**	**IEMs (% of PIND cases)**
Nunn et al. (6)^*^	80	63	50 (62.5%)
Keene et al. (5)	60	52	45 (75.0%)
Verity et al. (3)	548	362	141^**^
Devereux et al. (1)	598	577	135^***^
Verity et al. (2)	1259	1114	660^****^
Verity et al. (4)	2050	1925	675^*****^

* Excluded children with Rett disorder; 134 cases during study duration.

** Only ten most common diagnoses reported. IEMs represent 82.9% of the ten most common diagnoses.

*** Only five most common diagnoses reported. IEMs represent 82.3% of the five most common diagnoses.

**** Only thirty most common diagnoses reported. IEMs represent 83.2% of the thirty most common diagnoses.

***** *Only ten most common diagnoses reported. IEMs represent 88.2% of the ten most common diagnoses*.

Interestingly, most of these IEMs represent storage disorders, but other categories are represented as well. In summary, more than half, 59% of the well-documented IEMs (34/57) were categorized in the group “storage disorders” mainly due to different types of MPS (type I, II, IIIA, IIIB, IIIC, IIID, VII) and NCL (type 1, 2, 3, 5, 6, 7, 8, 10, 12, 14) which represent 17 of the 34 included storage disorders (50%).

It is known that IEMs may present with stable DD/ID. Metabolic evaluation is advised in children with DD/ID and yields a diagnosis in 0.2–4.6% of tested children (17). In 2012, 81 IEMs presenting with intellectual disability were listed in the review of Karnebeek et al. (22). By 2018, this number had increased to more than 110 (14, 18, 19). Since 52% of the IEMs presenting with PIND (*n* = 44) can be detected through standard metabolic blood and urine tests and a growing number of IEMs are amenable to treatment. Leading to stabilization or reversal of deterioration, early diagnosis of IEMs is advantageous. In addition, the correct diagnosis of IEMs will aid in accurate care of the child, through management of symptoms or co-morbidities and genetic counseling including assessment of prognosis to the family. Diagnosis of IEMs is becoming more accessible through exome and genome sequencing. Its place in the diagnostic work-up varies according to local availability and clinical practice as well as the patient's phenotype; the yield of exomes in metabolic and neurogenetic phenotypes varies form 16–68% (20, 21). Metabolic testing has the advantage of a fast turn-around time, relative affordability and specificity and can validate exome findings or point toward the affected pathway.

Treatment such as vitamin supplementation or dietary management may lead to halt of progression or reversal of symptoms caused by IEMs. An example is hydroxocobalamin in cobalamin C deficiency (MIM#277400), where supplementation of hydroxocobalamin stabilizes clinical deterioration, prevents metabolic decompensation, and improves systemic manifestations. Biotinidase deficiency (MIM#253260) is another example of an IEM causing intellectual disability where treatment through biotin supplementation improves psychomotor development, neurological and systemic manifestations. Dietary restrictions range from dietary phenylalanine restrictions in patients with phenylketonuria (MIM#261600), protein restriction in patients with isovaleric acidemia (MIM#243500), citrullinemia (MIM#215700) and argininemia (MIM#207800), to a ketogenic diet as treatment for patients with GLUT1 deficiency syndrome (MIM#606777) and PDH complex deficiency (MIM#312170) (15). New therapeutic modalities for IEMs that may present with PIND are continuously under development, and treatments targeting the pathophysiology of IEMs are showing promising results. Cerliponase alfa (Brineura^TM^), a recombinant human tripeptidyl peptidase-1 (TPP1) is an example of a recently developed treatment for neuronal ceroid lipofuscinosis type 2 (NCL2, MIM# 204500). NCL2 presents with progressive intellectual and neurological deterioration in children due to tripeptidyl peptidase-1 (TPP1) deficiency which causes accumulation of ceroid lipopigments in cells. Infusion of cerliponase alfa into the cerebrospinal fluid has shown to significantly slow the rate of deterioration of motor and language function in children with NCL2 (22, 23). Another emerging therapeutic modality is hematopoietic stem cell gene therapy (HSCGT). HSCGT which is currently under development for IEMS such as metachromatic leukodystrophy (MIM#250100) (24), X-linked adrenoleukodystrophy (MIM#300100) (25), Canavan disease (MIM#271900) (26, 27), mucopolysaccharidosis (MPS type I, II, IIIA, IIIB, VI) (28, 29) and neuronal ceroid lipofuscinosis (NCL type 1, 2, 3, 5, 6, 10, and 11) (30). The possibility of treating IEMs and thereby halting or reversing symptoms enhances the demand of an expeditious diagnostic process to ensure timely treatment in children presenting with symptoms caused by IEMs.

## Limitations

Progressive intellectual and neurological deterioration is a definition used by PIND research groups and in this review, but this definition is not used consistently throughout the medical literature. We tried to include all potential terms for PIND, and discussed this with experts specialized in metabolic diseases and an intellectual disability physician. However, it remains possible that some articles describe PIND with terms that were not included in the search. Therefore, this article should be perceived as a scope of the latest articles of PIND and not a full review.

Several of the included case reports described symptoms insufficiently, for example not mentioning frequency of seizures, leading to difficulties matching these symptoms with our definition of PIND. This contributed to the extensive list of IEMs with PIND in single cases or families, for example with IEMs like MERRF, Wilson's disease, thiamine 2 transporter deficiency, and vanishing white matter leucodystrophy.

An additional limitation of this review is the occurrence of seizures and metabolic strokes in IEMs. Neurocognitive regression or loss of skills appearing after seizures or metabolic strokes may be due to brain damage rather than a primary consequence of the IEMs. Since, in turn, seizures or metabolic strokes are caused by the underlying IEMs, these disorders were not excluded. When epilepsy or metabolic decompensation is recorded in IEMbase, the data is displayed in Supplemental Table S1. An example is the inclusion of MELAS, in which metabolic strokes are a characteristic feature, and thus PIND may be due to progressive brain damage caused by these strokes (10).

We are aware of the aforementioned limitations and acknowledge the risk of bias due to the low level of evidence in this review. Additional research on these rare IEMs and consultation of metabolic disease experts should decrease this risk.

## Conclusions

In summary, we present the first comprehensive overview of IEMs presenting with PIND, and provide a structured approach to diagnosis and treatment. Clearly IEMs constitute the largest group of genetic PIND conditions and have the advantage of detectable biomarkers as well as amenability to treatment. Thus, the clinician should keep IEMs at the forefront of the diagnostic workup of a child with PIND. With the ongoing discovery of new IEMs, expanded phenotypes, and novel treatment strategies, continuous updates to this work will be required. We invite colleagues from around the world to provide input on this topic which will enable us to practice precision medicine, optimizing clinical care and outcomes for these individuals and their families.

## Data Availability Statement

Publicly available datasets were analyzed in this study. This data can be found here: https://www.ncbi.nlm.nih.gov/pubmed/; http://www.iembase.org/; http://treatable-id.org/.

## Author Contributions

HW, ET-F, and LT performed the literature review, selected articles and extracted data, prepared the 1st draft of manuscript and tables (all under subversion). JL extracted data on each IEM from diverse research sources and contributed to the tables and manuscript. AE contributed to the systematic search, supervision of co-authors and editing of the manuscript. CF and CK designed the study, supervised the work of the co-authors and drafted the manuscript and made edits. All authors critically read, revised, and approved the current manuscript.

### Conflict of Interest

The authors declare that the research was conducted in the absence of any commercial or financial relationships that could be construed as a potential conflict of interest.

## Footnotes

^1^OMIM® and Online Mendelian Inheritance in Man® are registered trademarks of the Johns Hopkins University. www.omim.org.
