# Erratum: A Scoping Review of Inborn Errors of Metabolism Causing Progressive Intellectual and Neurologic Deterioration (PIND)

**DOI:** 10.3389/fneur.2020.00884

**Published:** 2020-07-28

**Authors:** Frontiers Production Office

**Affiliations:** Frontiers Media SA, Lausanne, Switzerland

**Keywords:** PIND, inherited metabolic diseases, neurodegeneration, dementia, loss of skills, genetic, treatment, diagnosis

Due to a production error, the incorrect **Supplementary Material** was published online. The publisher apologizes for this mistake.

The original article has been updated.

