# Supplementary material for: A Scoping Review of Inborn Errors of Metabolism Causing Progressive Intellectual and Neurologic Deterioration (PIND)
Source: Front Neurol. 2020 Feb 18;10:1369. doi: 10.3389/fneur.2019.01369 (PMC7040240; doi:10.3389/fneur.2019.01369)
Supplement: Supplementary file 1 [file Table_3.DOCX]

|  | |  |  |  |  |  |
| --- | --- | --- | --- | --- | --- | --- |
| **Supplemental Table S1.** IEMs presenting with PIND. | |  |  |  |  |  |
| **IEM name** | **NBS** | | |  |  |  |
| **A. DISORDERS OF NITROGEN-CONTAINING COMPOUNDS** | | |  |  |  |  |
| Adenylosuccinate lyase deficiency* |  | | |  |  |  |
| Aicardi-Goutieres^*^ |  | | |  |  |  |
| **Arginase 1 deficiency*** | + USA | | |  |  |  |
| Beta-alanine alpha-ketoglutarate transaminase deficiency* |  | | |  |  |  |
| **CAD deficiency** | + NL, USA | | |  |  |  |
| Canavan disease |  | | |  |  |  |
| **Carbamoylphosphate synthetase I deficiency*** |  | | |  |  |  |
| **Glutaric aciduria type 1** | + EN, DE, NL, USA | | |  |  |  |
| NBS: Newborn screening *Single cases PIND associated IEMs  CSF: Cerebrospinal fluid Treatable IEMS are in **bold** lettering  EM: Electron microscopy Characteristic findings are in **bold** lettering  ERG: Electroretinography  MRI: Magnetic resonance imaging | | | |  |  |  |
|  | | | | |  | |

|  |  |  |
| --- | --- | --- |
|  | |  |
| **IEM name** | **NBS** | |
| **A. DISORDERS OF NITROGEN-CONTAINING COMPOUNDS** | | |
| **Guanidinoacetate methyltransferase (GAMT) deficiency*** |  | |
| **HSD10 deficiency** |  | |
| **Hyperornithinemia-hyperammonemia-homocitrullinemia syndrome*** |  | |
| **Maple syrup urine disease (MSUD) ^*^** | + EN, DE, NL, USA | |
| **Methylglutaconic aciduria type I*** |  | |
| Nonketotic hyperglycinemia* |  | |
| NBS: Newborn screening *Single cases PIND associated IEMs  CSF: Cerebrospinal fluid Treatable IEMS are in **bold** lettering  EM: Electron microscopy Characteristic findings are in **bold** lettering  ERG: Electroretinography  MRI: Magnetic resonance imaging | | |

| **IEM name** | | **NBS** | |
| --- | --- | --- | --- |
| **A. DISORDERS OF NITROGEN-CONTAINING COMPOUNDS** | | | |
| Sulfite oxidase deficiency |  | | |
| **B. DISORDERS OF VITAMINS, COFACTORS, METALS AND MINERALS** | | |  |
| **Biotinidase deficiency** | | + DE, NL | |
| **Folate receptor-alfa deficiency** | |  | |
| Hereditary folate malabsorption* | |  | |
| IBA57 deficiency* | |  | |
| Leukoencephalopathy associated with APOA1BP gene mutations* | |  | |
| NBS: Newborn screening *Single cases PIND associated IEMs  CSF: Cerebrospinal fluid Treatable IEMS are in **bold** lettering  EM: Electron microscopy Characteristic findings are in **bold** lettering  ERG: Electroretinography  MRI: Magnetic resonance imaging | | | |

| **IEM name** | **NBS** |  |
| --- | --- | --- |
| **B. DISORDERS OF VITAMINS, COFACTORS, METALS AND MINERALS** | |  |
| **Methylenetetrahydrofolate reductase deficiency** |  |  |
| **Menkes disease** |  |  |
| **Methylcobalamin deficiency, cblE type*** |  |  |
| **Methylmalonic aciduria and homocystinuria, cblC type** | + NL, USA |  |
| **Molybdenum cofactor deficiency** |  |  |
| Thiamine transporter 2 deficiency* |  |  |
| Neurodegeneration with brain iron accumulation (NBIA) |  |  |
| Beta-propeller protein-associated neurodegeneration (BPAN) |  |  |
| Pantothenate kinase-associated neurodegeneration (PKAN) |  |  |
| NBS: Newborn screening *Single cases PIND associated IEMs  CSF: Cerebrospinal fluid Treatable IEMS are in **bold** lettering  EM: Electron microscopy Characteristic findings are in **bold** lettering  ERG: Electroretinography  MRI: Magnetic resonance imaging | |  |

| **IEM name** | **NBS** | | |
| --- | --- | --- | --- |
| **B. DISORDERS OF VITAMINS, COFACTORS, METALS AND MINERALS** | | |  |
| Wilson disease* |  | | |
| **C. DISORDERS OF CARBOHYDRATES** | |  |  |
| Pompe disease |  | | |
| **D. MITOCHONDRIAL DISORDERS OF ENERGY METABOLISM** | |  |  |
| Combined Oxidative Phosphorylation Defect 6* |  | | |
| Costeff syndrome |  | | |
| **COQ2 deficiency*** |  | | |
| **Glucose transporter deficiency^*^** |  | | |
| Leber Hereditary Optic Neuropathy, LHON* |  | | |
| Myoclonic Epilepsy associated with Ragged Red Fibers (MERRF)* |  | | |
| TWINKLE mitochondrial DNA helicase deficiency* |  | | |
| L-2-hydroxyglutaric aciduria |  | | |
| Leigh syndrome |  | | |
| **Mitochondrial Myopathy, Encephalopathy, Lactic Acidosis and Stroke-like episodes (MELAS)** |  | | |
| NBS: Newborn screening *Single cases PIND associated IEMs  CSF: Cerebrospinal fluid Treatable IEMS are in **bold** lettering  EM: Electron microscopy Characteristic findings are in **bold** lettering  ERG: Electroretinography  MRI: Magnetic resonance imaging | | | |

| **IEM name** | **NBS** | |
| --- | --- | --- |
| **D. MITOCHONDRIAL DISORDERS OF ENERGY METABOLISM** | |  |
| MitCHAP-60 disease |  | |
| OPA1 disease |  | |
| MEGDEL syndrome | + USA | |
| Mitochondrial depletion syndrome 4A |  | |
| **E. DISORDERS OF LIPIDS** |  | |
| **Cerebrotendinous xanthomatosis** |  | |
| NBS: Newborn screening *Single cases PIND associated IEMs  CSF: Cerebrospinal fluid Treatable IEMS are in **bold** lettering  EM: Electron microscopy Characteristic findings are in **bold** lettering  ERG: Electroretinography  MRI: Magnetic resonance imaging | | |

| **IEM name** | **NBS** | |
| --- | --- | --- |
| **E. DISORDERS OF LIPIDS** |  | |
| Neuronal ceroid lipofuscinosis type 1 |  | |
| **F. DISORDERS OF TETRAPYRROLES** | | |
| Crigler-Najjar syndrome^*^ |  | |
| **G. STORAGE DISORDERS** |  | |
| **Aspartylglucosaminuria** |  | |
| Farber disease* |  | |
| Fucosidosis |  | |
| **Gaucher disease** |  | |
| GM1 gangliosidosis |  | |
| GM2 gangliosidosis, AB variant |  | |
| **Krabbe disease** |  | |
| **Mannosidosis** |  | |
| **Metachromatic leucodystrophy** |  | |
| NBS: Newborn screening *Single cases PIND associated IEMs  CSF: Cerebrospinal fluid Treatable IEMS are in **bold** lettering  EM: Electron microscopy Characteristic findings are in **bold** lettering  ERG: Electroretinography  MRI: Magnetic resonance imaging | |  |

| **IEM name** | **NBS** |
| --- | --- |
| **G. STORAGE DISORDERS** |  |
| Mucolipidosis type 1* |  |
| Mucolipidosis type 4* |  |
| **Mucopolysaccharidosis type 1 (MPS I)** |  |
| **Mucopolysaccharidosis type 2 (MPS II)** |  |
| **Mucopolysaccharidosis type 3A (MPS IIIA)** |  |
| NBS: Newborn screening *Single cases PIND associated IEMs  CSF: Cerebrospinal fluid Treatable IEMS are in **bold** lettering  EM: Electron microscopy Characteristic findings are in **bold** lettering  ERG: Electroretinography  MRI: Magnetic resonance imaging | |

| **IEM name** | **NBS** |  |  |  |  |  |  |
| --- | --- | --- | --- | --- | --- | --- | --- |
| **G. STORAGE DISORDERS** | |  |  |  |  |  |  |
| **Mucopolysaccharidosis type 3B (MPS IIIB)** |  |  |  |  |  |  |  |
| **Mucopolysaccharidosis type 3C (MPS IIIC)** |  |  |  |  |  |  |  |
| **Mucopolysaccharidosis type 3D (MPS IIID)** |  |  |  |  |  |  |  |
| **Mucopolysaccharidosis type 7 (MPS VII)** |  |  |  |  |  |  |  |
| Multiple sulfatase deficiency |  |  |  |  |  |  |  |
| **Neuronal ceroid lipofuscinosis 2** |  |  |  |  |  |  |  |
| Neuronal ceroid lipofuscinosis 3 |  |  |  |  |  |  |  |
| NBS: Newborn screening *Single cases PIND associated IEMs  CSF: Cerebrospinal fluid Treatable IEMS are in **bold** lettering  EM: Electron microscopy Characteristic findings are in **bold** lettering  ERG: Electroretinography  MRI: Magnetic resonance imaging | |  |  |  |  |  |  |
|  |  |  |  |  |  |  |  |
|  |  |  |  |  |  |  |  |
|  |  |  |  |  |  |  |  |
|  |  |  |  |  |  |  |  |
|  |  |  |  |  |  |  |  |
|  |  |  |  |  |  |  |  |
|  |  |  |  |  |  |  |  |
|  |  |  |  |  |  |  |  |
|  |  |  |  |  |  |  |  |

| **IEM name** | **NBS** | |  |
| --- | --- | --- | --- |
| **G. STORAGE DISORDERS** | |  |  |
| Neuronal ceroid lipofuscinosis 5 |  | |  |
| Neuronal ceroid lipofuscinosis 6 |  | |  |
| Neuronal ceroid lipofuscinosis 7 |  | |  |
| Neuronal ceroid lipofuscinosis 8 |  | |  |
| Neuronal ceroid lipofuscinosis 10 |  | |  |
| Neuronal ceroid lipofuscinosis 12 |  | |  |
| NBS: Newborn screening *Single cases PIND associated IEMs  CSF: Cerebrospinal fluid Treatable IEMS are in **bold** lettering  EM: Electron microscopy Characteristic findings are in **bold** lettering  ERG: Electroretinography  MRI: Magnetic resonance imaging | | | |
|  |  |  |  |
|  |  |  |  |
|  |  |  |  |
|  |  |  |  |
|  |  |  |  |
|  |  |  |  |

| **IEM name** | **NBS** |
| --- | --- |
| **G. STORAGE DISORDERS** | |
| Neuronal ceroid lipofuscinosis 14 |  |
| Niemann-Pick type A |  |
|  |  |
|  |  |
|  |  |
|  |  |
|  |  |
| **Niemann-Pick disease type C1** |  |
|  |  |
|  |  |
|  |  |
|  |  |
|  |  |
| **Niemann-Pick disease type C2** |  |
|  |  |
|  |  |
| Salla disease |  |
|  |  |
|  |  |
|  |  |
|  |  |
|  |  |
|  |  |
| Sandhoff disease |  |
|  |  |
|  |  |
|  |  |
|  |  |
|  |  |
| Tay-Sachs disease |  |
|  |  |
|  |  |
|  |  |
| NBS: Newborn screening *Single cases PIND associated IEMs  CSF: Cerebrospinal fluid Treatable IEMS are in **bold** lettering  EM: Electron microscopy Characteristic findings are in **bold** lettering  ERG: Electroretinography  MRI: Magnetic resonance imaging | |

| **IEM name** | | **NBS** | | | | |  |  |  |  |  |  |  |  |  |  |  |
| --- | --- | --- | --- | --- | --- | --- | --- | --- | --- | --- | --- | --- | --- | --- | --- | --- | --- |
| **H. DISORDERS OF PEROXISOMES AND OXALATE** | | | | | | |  |  |  |  |  |  |  |  |  |  |  |
| X-linked adrenoleukodystrophy | |  | | | | |  |  |  |  |  |  |  |  |  |  |  |
| Zellweger spectrum disorders | |  | | | | |  |  |  |  |  |  |  |  |  |  |  |
| **I. CONGENITAL DISORDERS OF GLYCOSYLATION** | | | | | | |  |  |  |  |  |  |  |  |  |  |  |
| Congenital disorder of glycosylation, type 1a (CDG 1A)* |  | | | |  | | |  |  |  |  |  |  |  |  |  |  |
| **OTHER** | | | | | | |  | | |  |  |  |  |  |  | **Transaminase (plasma) (n-↑)** |  |
| eIF2B-related diseases* | | |  |  | |  |  |  |  |  |  |  |  |  |  |  |  |
| NBS: Newborn screening *Single cases PIND associated IEMs  CSF: Cerebrospinal fluid Treatable IEMS are in **bold** lettering  EM: Electron microscopy Characteristic findings are in **bold** lettering  ERG: Electroretinography  MRI: Magnetic resonance imaging | | | | | | |  | |  |  |  |  |  |  |  |  |  |
